# Learning curve for microscopic unilateral laminectomy for bilateral decompression surgery using the cumulative summation test for learning curve

**DOI:** 10.1097/MD.0000000000031069

**Published:** 2022-10-07

**Authors:** Jiwon Park, Hyun-Jin Park, Sang-Min Park, Jun-Young Choi, Ho-Joong Kim, Jin S. Yeom

**Affiliations:** a Department of Orthopedics, Korea University Ansan Hospital, Gyeonggi-do, Korea; b Department of Orthopedic Surgery, Spine Center, Kangnam Sacred Heart Hospital, Hallym University College of Medicine, Seoul, Korea; c Spine Center and Department of Orthopedic Surgery, Seoul National University College of Medicine and Seoul National University Bundang Hospital, Seongnam, Korea.

**Keywords:** learning curve, minimally invasive decompression, spinal stenosis, unilateral laminectomy bilateral decompression

## Abstract

A retrospective observational study The purpose of this study was to characterize the learning curve for a single level unilateral laminectomy and bilateral decompression in lumbar spinal stenosis using a learning curve cumulative summation test. Unilateral laminectomy and bilateral decompression for lumbar spinal stenosis proposes a potential benefit with minimizing surgery-related instability compared to traditional bilateral laminectomy, by preserving posterior stabilizing structures and contralateral facet joint and neural arch. Due to a narrow surgical corridor, it is considered to exhibit a steep learning curve as other types of minimally invasive procedures. However, there are few reports available regarding learning curve of unilateral laminectomy and bilateral decompression. The learning curve of a single surgeon performing single level unilateral laminectomy and bilateral decompression was assessed using learning curve cumulative summation test analysis. The surgeon had minimal experience in open decompressive laminectomy but no previous experience in unilateral laminectomy and bilateral decompression. Procedure success was defined as an operation time less than 75 minutes. Surgery related complications were recorded. Total 194 consecutive patients, who underwent primary single level unilateral laminectomy and bilateral decompression by a single spine surgeon, were included. The mean operative time for unilateral laminectomy and bilateral decompression was 64.6 ± 23.6 minutes. The mean operative time in the early learning period (≤29^th^ case) was 80.6 ± 20.9 minutes, and that in the late learning period (after 29^th^ case) was 61.8 ± 22.7 minutes, respectively. The overall complication rate was 13.9%. Majority of complications occurred in the early learning period. The learning curve cumulative summation test signaled competency for unilateral laminectomy and bilateral decompression at the 29^th^ operation, indicating that the surgeon reached the competent level. In addition, based on the cumulative summation test, the surgeon seemed to maintain his competency for the procedure. This study showed that surgical experience reduced the operation time and surgery related complications. For inexperienced surgeon to achieve an acceptable outcome in unilateral laminectomy and bilateral decompression, minimum 30 cases of unilateral laminectomy and bilateral decompression are required to reach competent level of surgery.

## 1. Introduction

Symptomatic lumbar spinal stenosis (LSS) is one of the most common surgical indications for spine surgery in elderly patients. As demand for LSS surgery has been grown, various different surgical techniques have been developed to treat LSS patients.^[1]^ Open decompressive laminectomy without fusion is a traditional surgical method for LSS, by which posterior structures including the lamina, spinous process, and ligamentum flavum, medial facet joint can be removed.^[1,2]^ Recently, unilateral laminectomy and bilateral decompression (ULBD) surgery is considered a viable option for the treatment of LSS since the popularity of minimally invasive surgery has increased.^[1–5]^ With its aim of preserving normal anatomy and minimizing surgical morbidity, the utility of ULBD has been widely accepted.

ULBD is a challenging procedure, mainly due to difficulty in identifying degenerative anatomical change within a narrow surgical corridor under a microscope.^[1,6,7]^ Accordingly, ULBD requires a certain amount of time for a young, inexperienced surgeon to become competent enough to perform the procedure efficiently and safely.^[6,7]^ Since ULBD is a widely performed procedure for the treatment of LSS, it is essential for an inexperienced surgeon to assess how much training under the supervision of expertise is needed to become competent in the procedure before providing his own practice.

The learning curve cumulative summation test (LC-CUSUM) is an analytical tool, which was specifically designed to focus on the learning period of a procedure.^[5,8–11]^ Quantitative and statistical process-control methods of LC-CUSUM monitor individuals’ medical performance during the learning period, and may help determine when an individual achieves a predefined competent level of medical performance.^[8–10]^ To date, only a few studies on the learning curve for ULBD have been reported, and to our best knowledge, there is no previous study reporting a learning curve of ULBD using a LC-CUSUM.^[6,12]^

The purpose of this study was to determine the learning curve of ULBD for LSS using a LC-CUSUM analysis and to provide information on how many cases of ULBD were required to achieve competency for performing the procedure efficiently and safely.

## 2. Materials and Methods

The institutional review boards of our hospital approved the design and protocol of the present retrospective study and waived written informed consent (approval no. H-1803-456-103).

### 2.1. Patient population

A cohort of 282 patients who underwent decompressive laminectomy by a single orthopedic surgeon (S.-M.P.) between April 2017 and June 2020 was assessed in this retrospective study. The operator had 1 year’s fellowship training, and during the fellowship period, the surgeon had an experience in open decompressive laminectomy but no experience in ULBD.

The medical records and preoperative radiographic images of all patients were reviewed. Included for this retrospective analysis were symptomatic patients who had undergone single level decompressive laminectomy using ULBD from L1 to S1. Among the 282 patients, 88 patients were excluded for the study. Specifically, 29 patients who were undergone open laminectomy with other level fusion, 7 patients with intradural tumor, 34 patients with multilevel ULBD, and 18 patients who were operated at sacrum or thoracic level were excluded. Accordingly, total 194 patients were included in this study.

Baseline demographic data including age, sex, height, weight, and body mass index (BMI) were collected. The operation-related data included the operation time, postoperative drainage, hospital stay, and complications.

### 2.2. Surgical technique

All operative procedures were performed under microscope as described previously.^[3]^ With the patient prone on a Wilson frame or on a Jackson table, the surgery level and the incision position were identified by a fluoroscopy. A small 3 cm of midline incision was made for unilateral approach to expose the surgery level. The approached side was determined by the patient’s symptom: the more symptomatic side was chosen as the approached side. If the patient symptom was similar bilaterally, the left side approach was chosen.

Under microscope, the inferior aspect of the superior lamina of the surgery level was identified. Dissection of lamina was started at the spinous process-lamina junction of upper level on the approach side using 3-mm matchstick headed high-speed burr and Kerrison punch. Once removal of the proximal lamina was done at the attachment site of the ligamentum flavum, the distal lamina and the hypertrophied ligamentum flavum were excised using Kerrison punch and a freer periosteal elevator with dural sac identification. Ipsilateral subarticular zone and lateral recess were examined for additional decompression.

**Figure 1.**
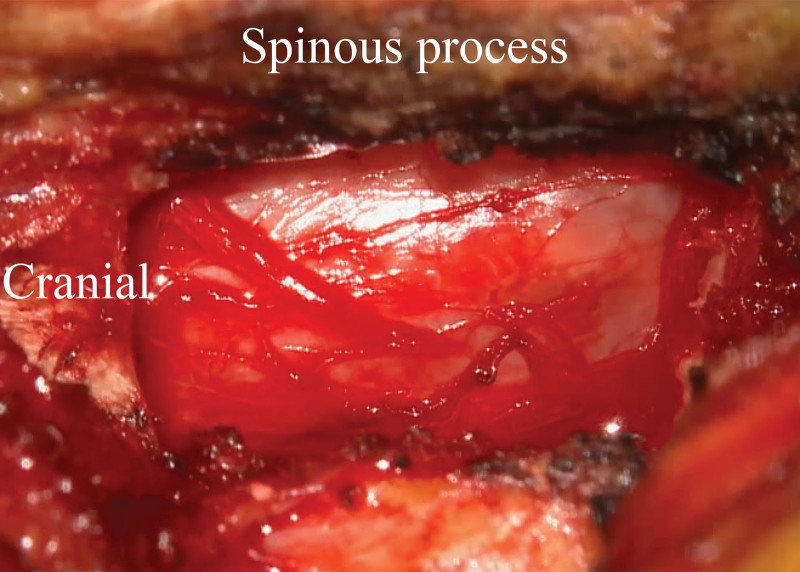
After well decompression, thecal sac as seen through a microscope.

After ipsilateral partial hemilaminectomy and decompression, undercutting of spinous process was performed for decompression of the contralateral side. The proximal lamina of the contralateral side was removed by high-speed burr and Kerrison punch. With dural sac identification, contralateral ligamentum flavum was removed completely for decompression. Contralateral subarticular zone and lateral recess were also assessed for additional decompression. Complete neural decompression was examined by dural pulsation. Bleeding control was achieved using bone. In every patient, drain was inserted before wound closure (Fig. 1).

### 2.3. Statistical analysis of LC-CUSUM

The LC-CUSUM was developed to analyze the learning curve by modifying CUSUM.^[8,13,14]^ When the trainee learns a new surgical technique, the status of training process is considered as “out of control” until the trainee reaches a proficient performance level, which considered as “in control.” The LC-CUSUM is a kind of control chart, which is a method of detecting a case that deviates from a predefined level by sequentially accumulating differences from a target value. In order to make LC-CUSUM graph, the unacceptable failure rate (ρ0), the failure rate (ρ1), the type I error, and the type II error must be determined. From these variables, the decision limit of h value can be calculated. The graph starts from “0” and does not penetrate “0.” For graphical representation, successful performance is represented by a downward slope on the graph, while inadequate performance (failure) is represented by an upward slope, but cannot penetrate “0.” The adequate performance level is considered acceptable when the LC-CUSUM score crosses this decision limit *h*. A standard CUSUM analysis was applied to the surgeon, once after the surgeon was considered as proficient level of performance.

In our study, the criteria for success of the learning curve were set to operation time. It was set as the operation time written on the anesthesia record, and this was defined the time from skin incision to skin closure time. The reference operation time was set to 75 minutes, which is the operation time of our senior professor (H.-J.K). Inadequate performance (failure) was defined as an operation time more than 75 minutes. We applied LC analysis according to previous literatures^[8,9,13–16]^ with the following parameters: the acceptable and unacceptable failure rates for “in control” and “out of control” processes, respectively, were a priori set at 20 and 40% by expert discussion in our department. These resulted in a decrease of 0.262 units for each successful measurement and an increase of 0.738 for each failure. With the optimizing type I error (0.05) and type II error, the decision limit h was set as -2.086. After LC-CUSUM analysis, standard CUSUM analysis was applied to the surgeon once his demonstrated adequate performance level. A decision limit h 2.524 was chosen for the CUSUM analysis. For calculating LC-CUSUM and CUSUM score, we used Excel software (Excel 2020, Microsoft, Redmond, WA). Other statistical analyses were performed using Stata/MP 15.0 (StataCorp LLC, College Station, TX). A 2-sided *P* value < 0.05 was considered to indicate statistical significance.

## 3. Results

Total 194 patients (men: 90, women: 104) were assessed for the LC-CUSUM for ULBD. The mean age of patients at the time of surgery was 70.7 years, with a range of 37 to 95 years. We investigated baseline characteristics including age, sex, height, weight, and BMI as well as the surgery levels. Patients’ diagnosis were as follows: 171 cases of degenerative central stenosis; 20 cases of infectious spondylitis with epidural abscess; 2 cases of spinal metastasis; and 1 case of osteoporotic compression fracture (Table 1).

**Table 1:** Demographic factors of the patients involved in this study.

Characteristic	Data
No. of patients	194
Mean age, years (range)	70.7 (37–95)
Men/women (n)	90/104
Mean height, cm (range)	158.9 (134.5–186.1)
Mean weight, kg (range)	62.7 (39.3–90.2)
Body mass index, kg/m^2^ (range)	24.7 (16.5–37)
Operative level (n)	
L1-2	6
L2-3	21
L3-4	68
L4-5	93
L5-S1	6

**Figure 2.**
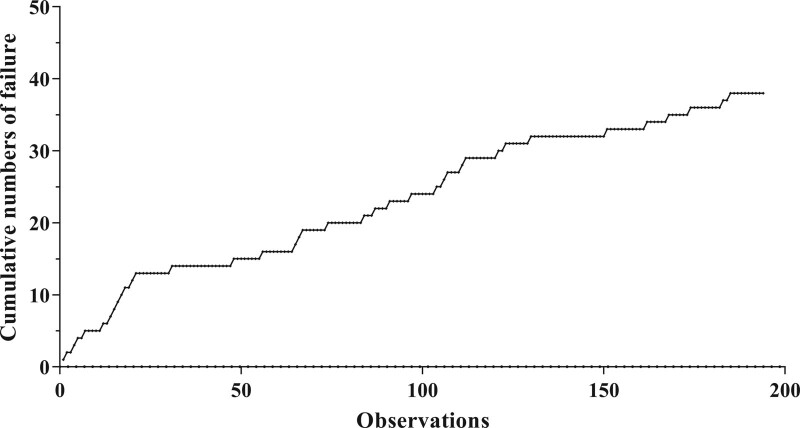
The cumulative number of failures for the surgeon was 38 surgeries during study period.

**Figure 3.**
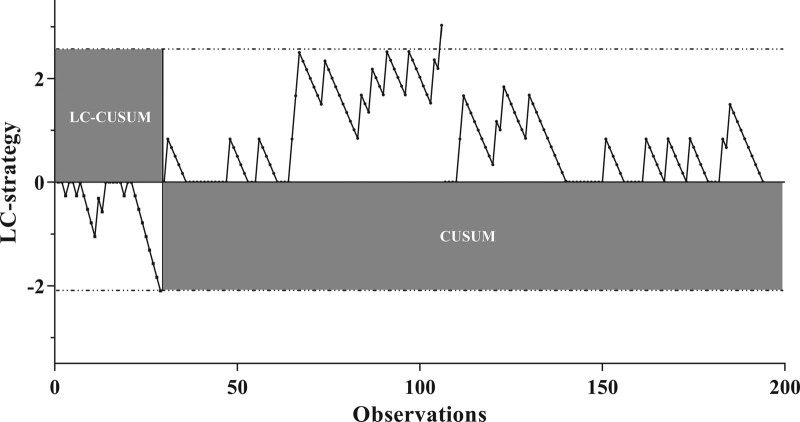
The LC-CUSUM analysis signaled competency after the 29^th^ operation. LC-CUSUM = learning curve cumulative summation test

The cumulative number of failures for the surgeon was 38 surgeries during study period (Fig. 2). The LC-CUSUM analysis signaled competency after the 29^th^ operation (Fig. 3). It means that the operator without previous experience in ULBD achieved surgical competency at the 29^th^ operation without supervision of expert.

A standard CUSUM analysis was applied after the 30^th^ surgery to determine if the surgeon retains a surgical proficiency. Throughout a standard CUSUM analysis, a total of 13 failures were found. And only one alarm was raised after the 106th surgery. No further alarms were raised until the observation ended.

The mean operative time for ULBD was 64.6 ± 23.6 minutes: 80.6 ± 20.9 minutes in the early learning period until 29^th^ case and 61.8 ± 22.7 minutes in the late learning period after 29^th^ case (Table 2). Table 2 listed the postoperative characteristics according to the learning period. There were no significant differences in postoperative drainage or hospital stay between the early learning period and the late learning period. While assessing the surgery-related complications, we found that majority of the complications occurred during the early learning period. Of 27 complications, dural tear accounted for the majority of complications. It occurred in a total of 23 cases: the occurrence of 27.6% in the early learning period until 29^th^ cases and 9% in the late learning period, respectively. There were 2 cases of hematoma occurred during the late learning period. During the early learning period, there were one case of incomplete decompression and one case of wrong level surgery. Overall, there were no serious surgery-related complications which required readmission or reoperation.

**Table 2:** Postoperative characteristics according to learning period.

Characteristic	Total	Early (≤29)	Late (>29)	*P* value*
Patients, n	194	29	165	
Operative time, min	64.6 ± 23.6	80.6 ± 20.9	61.8 ± 22.7	<0.001
Postoperative drainage, mL	65.4 ± 61.5	71.1 ± 59.1	64.4 ± 62.2	0.591
Hospital stay, d	5.2 ± 3.0	5.6 ± 2.5	5.1 ± 3.0	0.431
Complications, n (%)	27 (13.9%)	10 (34.5%)	17 (10.3%)	<0.001
Dural tear, n (%)	23 (11.9%)	8 (27.6%)	15 (9.1%)	
Hematoma, n (%)	2 (1.0%)	0 (0%)	2 (1.2%)	
Incomplete decompression, n (%)	1 (0.5%)	1 (3%)	0 (0%)	
Wrong level surgery, n (%)	1 (0.5%)	1(3%)	0 (0%)	
Surgical site infection	0 (0%)	0 (0%)	0 (0%)	

Numeric parameters are expressed as mean and standard deviation in parentheses. Categorical parameters are expressed as counts and percentages in parentheses.

* Statistical analyses were performed between early and late groups.

## 4. Discussion

Our study evaluated the learning curve for ULBD surgery in lumbar spine using LC-CUSUM analysis. LC-CUSUM analysis showed that ULBD required a substantial learning period of the inexperienced surgeon to become proficient in the procedure. For the inexperienced surgeon, at least 29 cases were required to reach a competent level of ULBD. In addition, a standard CUSUM analysis showed that the surgeon’s performance did not deviate from the acceptable predefined level of performance, and the surgeon maintained his competency thereafter.

ULBD is reported to have a steep learning curve for improvement of performance, especially for inexperienced surgeon due to its nature of minimally invasive surgery.^[2]^ Working within a small surgical corridor may result in difficulty identifying anatomy and accessing contralateral side.^[2]^ Compared to open bilateral decompression surgery, ULBD is considered to result in more significant dural sac retraction and a higher rate of intraoperative dural tear.^[17]^ These factors contributions may increase operation time and surgery-related complications for inexperienced surgeon. Therefore, learning a ULBD should be supervised until an appropriate level of performance has been reached.

Our study showed that the surgeon significantly reduced the operation time in the late learning period (after 29th case) compared to the early learning period (≤29^th^ case). There was significant difference found in overall surgery-related complication rates: 34.5% in the early learning period and 10.3% in the late learning period. A dural tear occurred in 27.6% of the patients in the early learning period while in 9.1% in the late learning period. There was one case of incomplete decompression, and one case wrong level surgery occurred in the early learning period. However, there was no significant difference found in the postoperative drainage amount and postoperative hospital stay. And there was no case for readmission or reoperation.

As many other studies did, the present study had several limitations. First, this study analyzed the learning curve for a single surgeon with a relatively small sample size. Since the time and the number of proceeds required for learning a new surgery varies widely depending on trainee and supervisor, on the kind of surgery, our finding may not be generalized. Second, other factors such as bleeding tendency, obesity (higher BMI), and severity of stenosis might have influenced the operation time as well as complication rates. Even though most of the patients in this study had degenerative central stenosis, overall diagnosis for ULBD was heterogeneous. Third, we used total operation time from skin incision to closure as a sole parameter for LC-CUSUM analysis. While ULBD is consisted of several steps such as ipsilateral laminectomy, ipsilateral and contralateral flavectomy, and bleeding control, it was impossible to measure how much time was required for each step due to the nature of retrospective study.

## 5. Conclusions

Our study showed a clear learning curve of ULBD performed by a single, inexperienced surgeon. LC-CULUM analysis shows initially high surgery-related complication rate, but it tends to decrease with increasing experience. Therefore, we advocate ensuring competent supervision of experienced surgeon for new surgeons at least during the first 30 procedures of ULBD. We believe that this finding may be helpful for those who contemplates an ULBD into their practice.

## Acknowledgment

We would like to thank Editage (www.editage.co.kr) for the English language editing.

## Author contributions

**Conceptualization:** Jiwon Park, Sang-Min Park.

**Data curation:** Jiwon Park, Jun-Young Choi, Sang-Min Park.

**Formal analysis:** Sang-Min Park.

**Investigation:** Jun-Young Choi, Sang-Min Park.

**Methodology:** Jun-Young Choi, Sang-Min Park.

**Project administration:** Sang-Min Park.

**Resources:** Sang-Min Park.

**Software:** Sang-Min Park.

**Supervision:** Ho-Joong Kim, Hyun-Jin Park, Jin S. Yeom.

**Validation:** Hyun-Jin Park, Jun-Young Choi.

**Visualization:** Hyun-Jin Park, Jun-Young Choi.

**Writing – original draft:** Jiwon Park, Sang-Min Park.

**Writing – review & editing:** Ho-Joong Kim, Hyun-Jin Park, Jin S. Yeom.
